# Dual Role of Triptolide in Interrupting the NLRP3 Inflammasome Pathway to Attenuate Cardiac Fibrosis

**DOI:** 10.3390/ijms20020360

**Published:** 2019-01-16

**Authors:** Xi-Chun Pan, Ya Liu, Yan-Yan Cen, Ya-Lan Xiong, Jing-Mei Li, Yuan-Yuan Ding, Yang-Fei Tong, Tao Liu, Xiao-Hong Chen, Hai-Gang Zhang

**Affiliations:** Department of Pharmacology, College of Pharmacy, Army Medical University (Third Military Medical University), Chongqing 400038, China; xichunpan@tmmu.edu.cn (X.-C.P.); liuya1979@hotmail.com (Y.L.); cenyanyan@163.com (Y.-Y.C.); xyl11594@sina.com (Y.-L.X.); Jingmei382@163.com (J.-M.L.); dingyy@yaopharma.com (Y.-Y.D.); tongyangfei@gmail.com (Y.-F.T.); cedarliu@foxmail.com (T.L.); pharma821@163.com (X.-H.C.)

**Keywords:** triptolide, cardiac fibrosis, inflammasome, NOD-like receptor protein 3, apoptosis-associated speck-like protein containing a CARD

## Abstract

In a previous paper, we reported that triptolide (TP), a commonly used immunomodulator, could attenuate cardiac hypertrophy. This present study aimed to further explore the inhibition of cardiac fibrosis by TP and the possible mechanism from the perspective of the NOD-like receptor protein 3 (NLRP3) inflammasome. Hematoxylin-eosin and Masson’s staining, immunohistochemistry, and immunofluorescence were performed to observe cardiac fibrotic changes in mice and mouse cardiac fibroblasts (CFs). The Western blot, colocalization, and immunoprecipitation were applied to detect protein expression and interactions. Results suggested that TP dose-dependently inhibited cardiac fibrosis induced by isoproterenol and collagen production of CFs induced by angiotensin II. TP exhibited an antifibrotic effect via inhibiting activation of the NLRP3 inflammasome, which sequentially decreased IL-1β maturation, myeloid differentiation factor 88 (MyD88)-related phosphorylation of c-Jun N-terminal kinase (JNK), extracellular regulated protein kinase 1/2 (ERK1/2), and TGF-β1/Smad signaling, and ultimately resulted in less collagen production. Moreover, TP showed no antifibrotic effect in *Nlrp3*-knockout CFs. Notably, TP inhibited the expression of NLRP3 and apoptosis-associated speck-like proteins containing a caspase recruitment domain (ASC) as well as inflammasome assembly, by interrupting the NLRP3-ASC interaction to inhibit inflammasome activation. Finally, TP indeed inhibited the NLRP3-TGFβ1-Smad pathway in vivo. Conclusively, TP was found to play a dual role in interrupting the activation of the NLRP3 inflammasome to attenuate cardiac fibrosis.

## 1. Introduction

Cardiac fibrosis, an irreversible pathophysiological process that contributes to nearly all types of heart disease, is characterized by excessive deposition of the extracellular matrix (ECM) comprised primarily of fibrillar collagen in the myocardial interstitium [1]. Cardiac fibroblasts (CFs) are responsible for producing ECM proteins, especially collagen, which provides a structural skeleton for cardiocytes and mediates electric conduction [2]. However, excessive accumulation of ECM results in structural and functional abnormalities of the heart, including stiffer ventricle and both systolic and diastolic dysfunction [3]. Therefore, it is crucial to elucidate the pathological mechanisms and develop new effective drugs for cardiac fibrosis.

It is well-known that immune regulation and inflammation participate in cardiac remodeling, including fibrosis [4]. In the past decade, NOD-like receptor protein 3 (NLRP3), a cytoplasmic pattern recognition receptor (PRR), was reported to be associated with the pathogenesis of cardiac fibrosis [5,6]. NLRP3 recognizes a wide variety of pathogen-derived or non-microbial alarm signals, such as pathogen/damage-associated molecular patterns (PAMPs/DAMPs), and leads to an inflammatory response in cardiac fibrosis [7]. Activated NLRP3 proteins form an oligomeric architecture known as NLRP3 oligomers, and then sequentially recruit apoptosis-associated speck-like protein containing a caspase recruitment domain (ASC) and pro-caspase-1, thereby forming the NLRP3 inflammasome, which can activate pro-caspase-1 to generate active caspase-1 [8]. Caspase-1 cleaves pro-IL-1β and pro-IL-18 into their active forms, IL-1β and IL-18, which activate the mitogen-activated protein kinase (MAPK) pathway to upregulate the expression of transforming growth factor β1 (TGF-β1) as well as other profibrotic genes, and induce fibroblast differentiation and collagen production [9]. Therefore, the NLRP3 inflammasome is expected to be a novel therapeutic target to develop safe therapies for the treatment of cardiac fibrosis [5].

Our previous studies have demonstrated that triptolide (TP), a diterpenoid ingredient isolated from the Chinese traditional herb *Tripterygium wilfordii*, could attenuate cardiac hypertrophy induced by isoproterenol (Iso) in mice [10]. We also found that TP might possess an anti-fibrotic effect based on MASSON staining data. Another group reported that TP could also alleviate Iso-induced cardiac remodeling in rats [11]. Moreover, two other groups suggested that TP inhibited cardiac fibrosis in a rat pressure overload model or diabetic cardiomyopathy model [12,13,14]. These findings strongly prove that TP can ameliorate cardiac fibrosis, but it is difficult to distinguish between cell types and how the underlying mechanism contributes to the activity of TP based on in vivo data. Collectively, the role of TP in attenuating cardiac fibrosis and the detailed mechanisms of this effect are still poorly understood. In this study, we aimed to evaluate the antifibrotic effect of TP in a mouse model of cardiac fibrosis, and further reveal its molecular mechanism combined with insights into the NLRP3 inflammasome pathway.

## 2. Results

### 2.1. Triptolide Attenuates Isoproterenol-Induced Cardiac Fibrosis and Collagen Deposition in Mice

**Figure 1:**
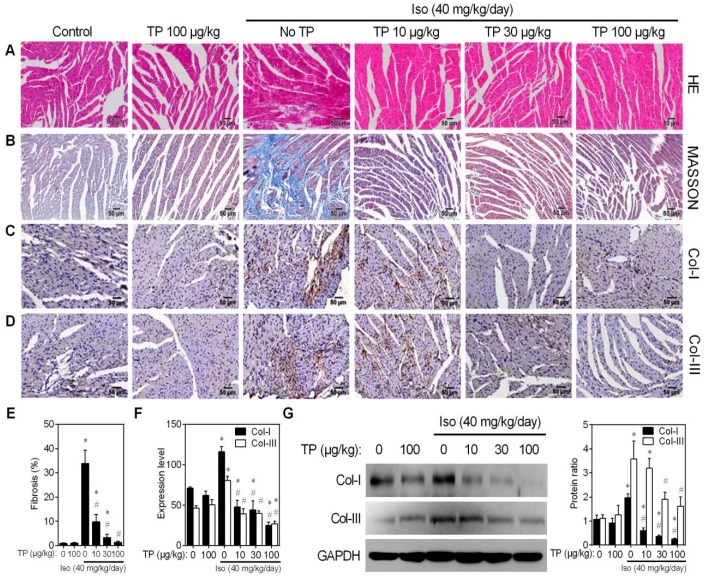
Triptolide attenuates cardiac fibrosis in mice. Cardiac fibrosis was induced by continuous Iso infusion (40 mg/kg/day for 14 days; s.c.) in mice (*n* = 5). Mice were treated with normal saline (control), Iso and/or triptolide (10, 30, 100 μg/kg) for 14 days, respectively. Cross-sections of the mid-ventricle were used for subsequent assays. (**A**) Hematoxylin-eosin (HE) staining. (**B**) Masson staining. (**C**–**D**) Immunohistochemistry (IHC) assays of collagen I/III (Col-I/III). Bar = 50 μm. (**E**) Fibrosis score. (**F**) Expression of Col-I/III in the mid-ventricle. (**G**) Western blot analysis of Col-I/III expression in mid-ventricle tissue lysate. Histograms represent protein ratios normalized to GAPDH (*n* = 3). * *p* < 0.01 vs. control; # *p* < 0.01 vs. Iso.

During the in vivo experiments, no mortality was observed in any treatment group. Hematoxylin-eosin (HE) staining was performed to observe histological alterations in the mouse ventricle. The results showed that Iso induced disarray of myofibrils and increased the ECM, which is mainly composed of collagen I and III (Col-I/III). Although TP alone had no effect on tissue morphology, it remarkably lessened the Iso-induced morphological changes (Figure 1A). Masson’s trichrome staining was applied to detect morphological changes in collagen that were indicative of fibrosis development. Ventricle tissue in the Iso group showed massive and intense collagen accumulation (blue) within the disrupted myocardium. However, TP alleviated cardiac fibrosis induced by Iso in a dose-dependent manner (Figure 1B,E). Consistently, immunohistochemistry (IHC) staining also indicated that TP displayed dose-dependent inhibition of Iso-induced Col-I and Col-III expression in the left ventricle (Figure 1C,D,F). However, there was no significant change in Col-I/Col-III ratio between groups. Finally, Western blot confirmed that TP could inhibit Iso-induced Col-I and Col-III expression in the total lysate of heart tissue (Figure 1G). Together, these results indicate that TP attenuates Iso-induced cardiac fibrosis in mice.

### 2.2. Triptolide Reduces Angiotensin-II-Induced Collagen Synthesis of CFs

**Figure 2:**
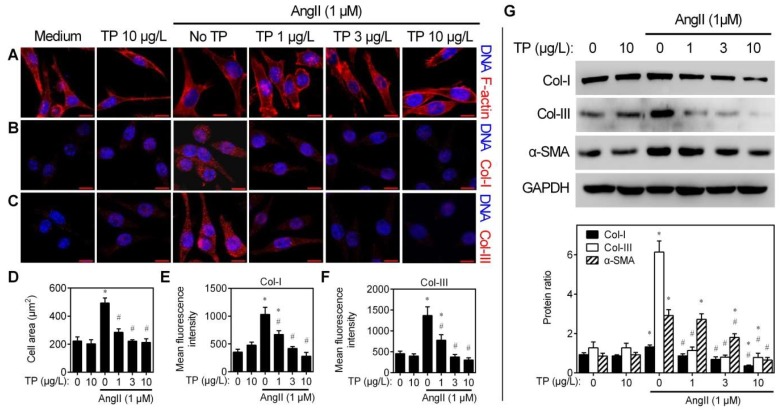
Triptolide inhibits collagen production in vitro. Cardiac fibroblasts (CFs) grown in plates were treated with AngII (1 μM) and/or TP (1, 3, 10 μg/L) for 24 h. (**A**) Cytoskeleton staining by rhodamine-phalloidin (acts on F-actin). (**B–C**) Immunofluorescence staining of Col-I/III. (**D**) Cell size (*n* = 50). (**E**–**F**) Expression of Col-I and Col-III in CFs (*n* = 50). (**G**) Western blot analysis of the expression of Col-I, Col-III, and α-smooth muscle actin (α-SMA) in CFs. Histograms represent the protein ratio normalized to GAPDH (*n* = 3). Bar = 10 μm. * *p* < 0.01 vs. medium; # *p* < 0.01 vs. AngII.

CFs play a crucial role in the accumulation of ECM by transforming into myofibroblasts and secreting collagen [15]. Therefore, CFs were used to evaluate the influence of TP on angiotensin II (AngII)-induced alterations. Firstly, cytoskeleton assays indicated that AngII increased the size of CFs, but those were dose-dependently inhibited by TP (1–10 μg/L) (Figure 2A,D). This data suggests that TP may inhibit myofibroblast differentiation of CFs. Secondly, immunofluorescence staining was performed to determine whether TP affects collagen production by CFs. Consistent with the IHC results presented in Figure 1, TP could dose-dependently inhibit AngII-induced Col-I and Col-III production within CFs (Figure 2B,C,E,F). Finally, similar results were seen by Western blot analysis (Figure 2G). TP also could inhibit AngII-induced production of α-smooth muscle actin (α-SMA), a marker of myofibroblast, further indicating that TP indeed inhibits myofibroblast differentiation of CFs (Figure 2G). The above results demonstrate that TP possesses antifibrotic activity in vitro.

### 2.3. Triptolide Reduces Collagen Production by Inhibiting TGF-β1/Smad Signaling

**Figure 3:**
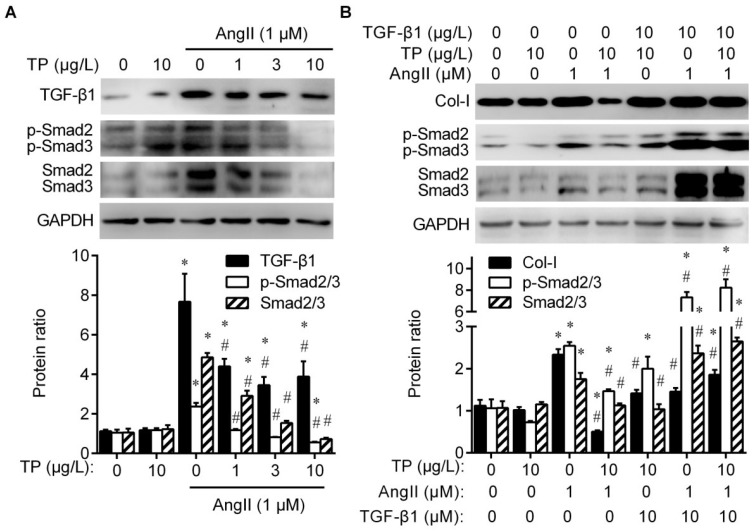
The TGF-β1/Smad pathway is crucial for the triptolide-mediated inhibition of collagen production in cardiac fibroblasts. (**A**) Cells were treated as described in Figure 2. The cell lysate was analyzed via Western blot with antibodies against TGF-β1, Smad2/3, and phospho-Smad2^Ser465/467^/3^Ser423/425^ (p-Smad2/3). (**B**) Cells grown in dishes were treated with AngII (1 μM) and TP (10 μg/mL), alone or in combination and with or without TGF-β1 (10 μg/mL). The cell lysate was analyzed by Western blot with antibodies against Col-I, Smad2/3, and p-Smad2/3. Histograms represent the protein ratio normalized to GAPDH (*n* = 3). * *p* < 0.01 vs. medium; # *p* < 0.01 vs. AngII.

TGF-β1/Smad signaling plays a critical role in collagen production in fibroblasts [1]. Therefore, the effects of AngII and TP on the protein levels of TGF-β1 and total/phosphorylated Smad2/3, the classical profibrotic factors, were examined by Western blot. The results showed that AngII significantly elevated the expression of TGF-β1, Smad2/3, and phospho-Smad2^Ser465/467^/3^Ser423/425^ (p-Smad2/3) in CFs; meanwhile, levels of these were dose-dependently inhibited by TP (1–10 μg/L; Figure 3A). To further confirm the effects of TP on TGF-β1/Smad signaling, TGF-β1 was employed to determine whether it could suppress TP activity. Herein, TP could also inhibit AngII-induced Col-I and p-Smad2/3 expressions, but TGF-β1 supplementation markedly reversed the inhibitory effect of TP (Figure 3B). These results suggest that TP reduces collagen production in CFs by inhibiting TGF-β1/Smad signaling.

### 2.4. Triptolide Inhibits Angiotensin II-Induced JNK and ERK1/2 Activation

**Figure 4:**
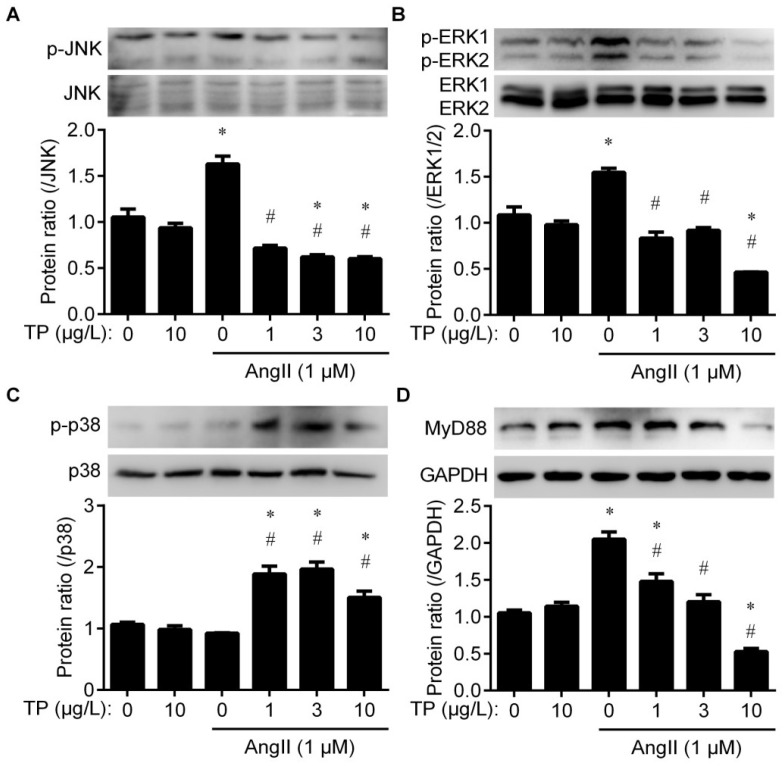
Triptolide inhibits ERK1/2 and JNK activation in cardiac fibroblasts. The cells were treated as described in Figure 2 and analyzed by Western blot with antibodies against (**A**) JNK and p-JNK, (**B**) ERK1/2 and p-ERK1/2, (**C**) p38 and p-p38, and (**D**) MyD88. Histograms represent the protein ratio, normalized to total JNK, total ERK, total p38, or GAPDH (*n* = 3), respectively. * *p* < 0.01 vs. medium; # *p* < 0.01 vs. AngII.

It is well-known that TGF-β1 expression is promoted by c-JUN activation, which is activated by the MAPK pathway [9]. Therefore, total and phosphorylated levels of p38, ERK1/2 (also known as P44/42) and JNK were detected to evaluate the influence of TP on the MAPK cascade in CFs during the fibrotic response. Western blot indicated that TP and AngII alone or in combination had no effect on the expression of total JNK; however, TP dose-dependently inhibited the AngII-induced accumulation of phospho-JNK^Thr183/Tyr185^ (p-JNK; Figure 4A; GAPDH band used as an internal reference is shown in Figure 4D). Consistently, TP and AngII had a similar effect on total ERK1/2 and phospho-ERK1/2^Thr202/Tyr204^ levels (p-ERK1/2; Figure 4B). TP or AngII alone had no effect on total p38 expression, but the combination treatment significantly facilitated the accumulation of phospho-p38^Thr180/Tyr182^ (p-p38; Figure 4C). In addition, MyD88, which is crucial for triggering IL-1R1-mediated MAPK activation [9], was also detected herein. The results indicated that TP and/or AngII had a similar effect on MyD88 expression, as on the expression of p-JNK and p-ERK1/2 (Figure 4D). These results suggest that TP inhibits TGF-β1/Smad signaling in CFs by decreasing the MyD88-mediated activation of JNK and ERK1/2.

### 2.5. Triptolide Reduces Collagen Production by Inhibiting NLRP3 Inflammasome Activation

**Figure 5:**
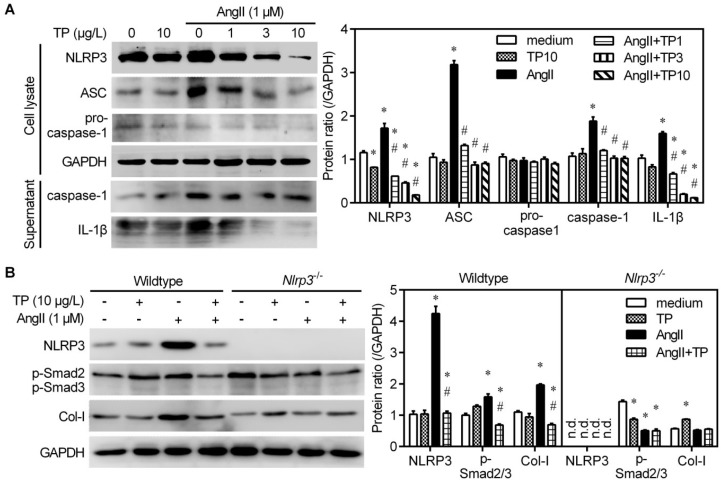
Triptolide downregulates NLRP3 inflammasome expression in cardiac fibroblasts (CFs). (**A**) Cells were treated as presented in Figure 2. Western blot was performed to detect the expression of NLRP3, apoptosis-associated speck-like proteins containing a caspase recruitment domain (ASC) and pro-caspase-1 in total cell lysate, and the release of caspase-1 and IL-1β in the supernatant. (**B**) CFs isolated from wildtype C57 mice or *Nlrp3*-knockout C57 mice (*Nlrp3*^−/−^) were treated with AngII (1 μM) and/or triptolide (TP; 10 μg/mL) for 24 h. Cell lysate was used to detect the expression of NLRP3, Col-I, and p-Smad2/3 by Western blot. Histograms represent the protein ratio normalized to GAPDH (*n* = 3). * *p* < 0.01 vs. medium; # *p* < 0.01 vs. AngII. n.d., not detected.

AngII facilitates NLRP3 inflammasome activation and induces the cleavage of pro-caspase-1 and the release of IL-1β and IL-18, thereby activating MAPK-TGFβ1-Smad signaling [16]. Thence, Western blot was performed to observe whether TP influences the expression of AngII-induced NLRP3 inflammasome components, including NLRP3, ASC, and pro-caspase-1, as well as mature caspase-1 and IL-1β. Under stimulation by AngII, the expression of NLRP3 and ASC was elevated in CFs. In addition, TP could dose-dependently inhibit AngII-induced NLRP3 and ASC expression, whereas it had no influence on pro-caspase-1 expression in total CF lysates (Figure 5A). Meanwhile, TP could dose-dependently reduce the AngII-induced accumulation of mature caspase-1 and IL-1β in the supernatant of CFs (Figure 5A). The above data suggest that TP inhibits AngII-induced NLRP3 inflammasome activation by downregulating the expression of NLRP3 and ASC.

To further confirm that the NLRP3 inflammasome participates in the antifibrotic effect of TP, *Nlrp3*-*knockout* (*Nlrp3*^−/−^) CFs were employed. In wildtype CFs (control), TP alone also significantly inhibited the expression of NLRP3 and Col-I, as well as the accumulation of p-Smad2/3 induced by AngII (left panel of Figure 5B). Simultaneously, in *Nlrp3*^−/−^ CFs, AngII could not increase Col-I and p-Smad2/3 accumulation, and TP lost the inhibition on AngII-induced pro-fibrotic changes (right panel of Figure 5B). Therefore, these results suggest that TP reduces AngII-induced collagen production by inhibiting activation of the NLRP3 inflammasome pathway.

### 2.6. Triptolide Interrupts Angiotensin II-Induced Formation of the NLRP3 Inflammasome

The above data indicate that TP downregulates NLRP3 and ASC expression to inhibit inflammasome activation. In addition to expression levels, NLRP3 or ASC protein oligomerization and the recruitment of ASC and caspase-1 by NLRP3 contribute to inflammasome activation [17,18]. Therefore, colocalization and immunoprecipitation (IP) assays were performed to observe whether TP affects NLRP3 or ASC oligomerization, or NLRP3-ASC or NLRP3-caspase-1 interactions.

**Figure 6:**
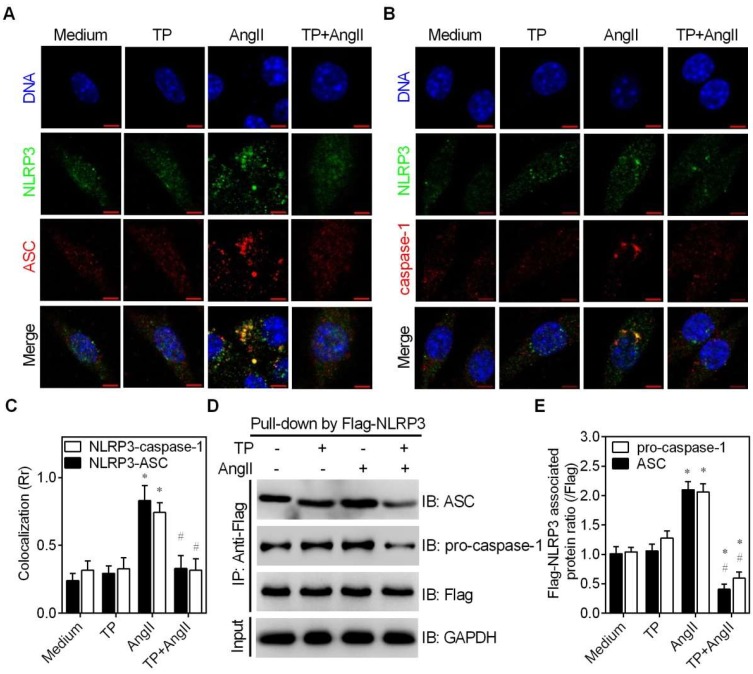
Triptolide inhibits NLRP3 inflammasome assembly in cardiac fibroblasts (CFs). (**A**) CFs were placed on glass slides and treated with AngII (1 μM) and/or triptolide (TP; 10 μg/mL) for 24 h. Cells were then stained for NLRP3 (Alexa Fluor 488; green) and ASC (Alexa Fluor 555; red) and observed under a laser confocal microscope; bar = 5 μm. (**B**) Experiments were performed as described in A, and the cells were stained for NLRP3 (Alexa Fluor 488; green) and caspase-1 (Alexa Fluor 555; red); bar = 5 μm. (**C**) Colocalization of NLRP3 with ASC or caspase-1, calculated using Pearson’s correlation coefficient (Rr) via ImageJ (*n* = 50). (**D**–**E**) Cells were transfected with a Flag-*Nlrp3* construct and treated as in A. Total lysate was used for immunoprecipitation (IP) using an anti-Flag antibody, and the associated pro-caspase-1 and ASC were detected via immunoblotting (IB). Histograms represent the protein ratio normalized to Flag (*n* = 3) * *p* < 0.01 vs. medium; # *p* < 0.01 vs. AngII.

Firstly, the confocal images of colocalization experiments showed that TP inhibited the AngII-induced expression of NLRP3 (green) and ASC (red), which was consistent with the above data obtained via Western blot analysis; however, TP alone or in combination with AngII could not induce the dispersion of NLRP3 or ASC dots (Figure 6A,B). These results suggest that TP has no significant influence on NLRP3 or ASC oligomerization.

Secondly, colocalization of NLRP3-ASC or NLRP3-caspase-1 in confocal images was observed by labeling NLRP3 with Alexa Fluor 488 (green) and labeling ASC or total caspase-1 with Alexa Fluor 555 (red). The results showed that AngII remarkably increased the number of yellow dots (merged green and red dots) within CFs and the Rr value calculated by ImageJ. However, TP significantly decreased the number of intracellular yellow dots and the Rr value (Figure 6A,C). These results indicate that TP inhibits AngII-induced NLRP3-ASC colocalization. Consistently, the results also indicated that TP similarly inhibited NLRP3-caspase-1 colocalization (Figure 6B,C). Considering that NLRP3 recruits ASC, which recruits pro-caspase-1, the above results suggest that TP affects NLRP3-ASC interactions to inhibit NLRP3-caspase-1 colocalization. Therefore, the above results strongly suggest that TP inhibits the recruitment of ASC by NLRP3.

Finally, to further investigate whether TP inhibits NLRP3 inflammasome assembly, IP experiments were performed to detect the influence of TP on the levels of NLRP3-associated ASC and caspase-1. Using an equal amount of Flag-NLRP3 as a control (pull-down with an anti-Flag antibody), AngII increased the protein levels of associated ASC and pro-caspase-1; however, these levels were significantly decreased in combination with TP (Figure 6D,E). Together, these results further indicate that the inhibition of AngII-induced NLRP3 inflammasome activation by TP is related to interrupting inflammasome assembly via inhibiting the NLRP3-ASC interaction.

### 2.7. Triptolide Inhibits Isoproterenol-Induced NLRP3-TGFβ1-Smad Signaling in the Mouse Ventricle

**Figure 7:**
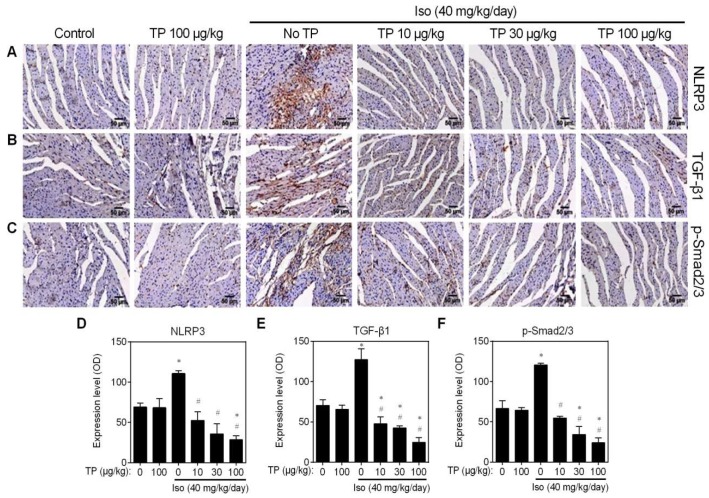
Triptolide inhibits NLRP3-TGFβ1-Smad signaling in the fibrotic ventricle of mice. Experiments were performed as presented in Figure 1. (**A**–**C**) IHC for NLRP3, TGF-β1 and p-Smad2/3; bar = 50 μm. (**D**–**F**) Fibrosis score. * *p* < 0.01 vs. control; # *p* < 0.01 vs. Iso.

The results obtained with the CFs in vitro indicate that TP inhibits AngII-induced collagen production by inhibiting the NLRP3-TGFβ1-Smad pathway. To further investigate the influence of TP on this pathway in vivo, IHC experiments were performed again using the same ventricular paraffin sections, as shown in Figure 1. The results showed that the NLRP3 protein level was significantly upregulated by Iso in the fibrotic area of the mouse’s left ventricle, but this expression level was dose-dependently decreased by TP (Figure 7A,D). Similarly, the same results for TGF-β1 and p-Smad2/3 were obtained (Figure 7B,C,E,F). These results further indicate that TP attenuates cardiac fibrosis by inhibiting the NLRP3-TGFβ1-Smad pathway.

## 3. Discussion

In this study, we reported that low-dose TP could attenuate mouse cardiac fibrosis induced by Iso (in vivo) or AngII (in vitro) via inhibition of the NLRP3 inflammasome-related profibrotic pathway through a dual role, by downregulating the expression of NLRP3 inflammasome components and by interrupting NLRP3 inflammasome assembly; together, these effects decrease the activation of the MAPK-TGFβ1-Smad pathway.

Unlike other organs and tissues, the adult heart possesses decreased regenerative potential after acute or chronic injury; instead, it repairs damaged tissue via fibrotic scar tissue that retains myocardial structural integrity. Importantly, it is well-known that pathological cardiac fibrosis contributes to nearly all forms of heart disease [2]. Cardiac fibrosis involves the deposition of types I and III collagen and ECM crosslinking; together, these processes result in altered compliance of myocardial fibers, stiffening of the ventricular wall, and impaired electrical conductance of the heart [19]. CFs are the essential cell type responsible for ECM homeostasis; however, under pathologic conditions such as heart injury, pressure overload, and neurohumoral factors, these cells may transform to myofibroblasts and promote cardiac fibrosis [20].

Although the pathophysiological stimuli leading to cardiac fibrosis differ in patients with various heart diseases, the cellular effectors that induce fibrotic alterations in CFs are highly similar [1,19]. Within these effectors, inflammation acts as the central platform that directly or indirectly promotes the expression of profibrotic genes, such as TGF-β1 [21]. In addition to sensing proinflammatory cytokine release from immune cells, CFs also possess PRRs, such as NLRP3, to recognize alarm signals and trigger an inflammatory response [22]. Numerous studies have indicated that the activation of NLRP3 in CFs can lead to the sequential recruitment of ASC and pro-caspase-1 form the NLRP3 inflammasome to induce maturation of IL-1β and IL-18, and finally result in TGF-β1-related cardiac fibrosis [5]. The present study clearly showed that stimulation with AngII or Iso increased the expression of NLRP3 and ASC during the process of fibrosis.

Previous studies have indicated that neurohumoral factors induce cardiovascular diseases through the NLRP3 inflammasome-related pathway [23,24]. However, the correlation between NLRP3 and neurohumoral factor-induced cardiac fibrosis, especially fibrotic changes in CFs, remains poorly understood. Herein, using wildtype and *Nlrp3*^−^^/−^ CFs, we found that AngII increased collagen production by increasing both NLRP3 expression and inflammasome assembly. Interestingly, the ability of AngII to induce collagen production was lost in *Nlrp3*^−^^/−^ CFs. Not surprisingly, the results of fibrosis induced by Iso infusion in vivo showed a similar trend. Our findings confirmed that neurohumoral factors could induce cardiac fibrosis through activating the NLRP3 inflammasome. It has been proven that *Nlrp3* transcription in immune cells is promoted by nuclear factor kappa B (NF-κB), a well-characterized nuclear factor [5]. Earlier, Lim et al. also reported that AngII facilitates angiotensin receptor-mediated reactive oxygen species (ROS) production to increase NF-κB activity and subsequent *Nlrp3* transcription in cardiocytes [23]. Therefore, we can deduce that AngII upregulates *Nlrp3* transcription by increasing NF-κB activity in CFs. Moreover, we also found that AngII could upregulate ASC, the platform molecule for the ASC-dependent inflammasome (NLRP3, AIM2 and Pyrin) [25]. This finding further suggests that AngII also promotes the activation of other types of ASC-dependent inflammasome.

As the main active compound of *T. wilfordii*, a Chinese herb widely used to treat autoimmune and inflammatory diseases, TP is a commonly used immunosuppressant in clinical practice. Numerous studies have indicated that TP exerts its anti-inflammatory effect by inhibiting NF-κB and downstream cytokines such as IL-1β, IL-6, and TNF-α in immune cells [26]. In studying cardiac fibrosis, several reports also suggested that TP functioned through inflammation-related pathways. Guo et al. reported that TP inhibited cardiac fibrosis via a toll-like receptor 4 (TLR4)-related pathway in a diabetic cardiomyopathy rat model [14]. Liu et al. and Zhang et al. respectively reported that TP attenuated Iso- or pressure overload-induced cardiac fibrosis in rats by inhibiting TGF-β1/Smad signaling [11,13]. Moreover, Zhang and colleagues further reported that TP inhibited NLRP3 expression in rat hearts, but whether it is related to the anti-fibrotic activity remains unclear [12]. Previously, our group also reported the similar anti-fibrotic effect of TP in a Iso-induced mouse model [10]. However, based on these in vivo results, it remains unclear that the anti-cardiac fibrosis activity of TP is related to which cell type of heart (cardiocytes, fibroblasts, and monocytes/macrophages, etc.), and whether TP functions through NLRP3 inflammasome. In this study, we confirmed that TP attenuated cardiac fibrosis in a mouse model, and further indicated that TP inhibits the fibrotic changes of CFs. Notably, we found that TP indeed functioned through the NLRP3 inflammasome pathway based on *Nlrp3*-knockout CFs and in vivo data.

Stimuli induce NLRP3 expression and oligomerization to form NLRP3 oligomers that in turn recruit ASC (also forms ASC oligomers) and pro-caspase-1, thereby resulting in inflammasome activation [27]. Therefore, inhibiting the expression of NLRP3 inflammasome components, decreasing NLRP3 or ASC oligomers formation, or interrupting NLRP3 inflammasome assembly would affect inflammasome activation. Although the structure of NLRP3 remains unclear, known NLRP3 inhibitors, such as MCC950 and β-hydroxybutyrate (BHB) inhibit the NLRP3 inflammasome by acting on ASC oligomerization [28]. In this study, we found that TP had no influence on NLRP3 or ASC oligomerization, but that it could inhibit NLRP3 and ASC expression, as well as NLRP3-ASC interactions, to inhibit inflammasome assembly. Therefore, TP can be defined as a novel NLRP3 inhibitor that downregulates NLRP3 expression and directly affects NLRP3-ASC assembly to inhibit inflammasome activation.

The present study has certain limitations. The downregulation of NLRP3 expression by TP may be related to inhibition of NF-κB activity, and the detailed mechanisms by which TP interrupts NLRP3-ASC interactions need to be further investigated. Nevertheless, with the safety record in anti-inflammatory applications, TP is suggested to be an alternative option for cardiac fibrosis via targeting the NLRP3 inflammasome.

## 4. Materials and Methods

### 4.1. Animals and Treatment

Adult male C57 mice (4–6 weeks old, weighing 18–22 g) and *Nlrp3*^−/−^ C57 mice (4–6 weeks old, weighing 18–22 g) were obtained from the Experimental Animal Center of our university. All animal experiments were performed according to the National Guidelines for Animal Care and Use (NIH Publication No. 85–23, revised 1996) and were approved by the Ethics Committee for Animal Experimentation of our university. Mice were employed to observe the influence of TP (10, 30, or 100 mg/kg) on cardiac fibrosis induced by infusion of Iso (40 mg/kg/day; s.c.; Sigma, St. Louis, MO, USA) for 14 days as described previously [10]. The mid-ventricle was stored in liquid nitrogen for Western blot or fixed with a neutral formalin solution and embedded in paraffin for histological assays.

### 4.2. Cell Culture

The tissue-block culture method was used to isolate mouse primary CFs from the left ventricle of wildtype C57 or *Nlrp3*^−/−^ C57 mice. Briefly, the ventricles were washed with PBS (50 μM; pH 7.2), minced and placed in dishes for a few minutes. The adherent pieces were retained and cultured in DMEM (Gibco; Grand Island, NY, USA) with 10% (*v*/*v*) FBS (Gibco), penicillin (100 U/mL), and streptomycin (100 μg/mL) for 10 days. After CFs grew from the border, tissue pieces were removed, and the cells were cultured in complete DMEM for subsequent tests. Herein, AngII (; 1 μM; Sigma) was used to induce collagen production in vitro.

### 4.3. Histological Observations

Paraffin sections (5 μm) were stained with HE or Masson’s trichrome as reported previously [10]. For IHC assays, primary antibodies (Cell Signaling; Beverly, MA, USA) against Col-I/III, NLRP3, TGF-β1, and phospho-Smad2^Ser465/467^/3^Ser423/425^ were used at a dilution of 1:200 according to the user manuals. The subsequent procedure was performed as previously reported [10]. Briefly, after incubation with a secondary antibody at room temperature, the slides were stained with 3,3′-diaminobenzidine tetrahydrochloride (DAB). After immersing the slides in distilled water to stop the reaction, the sections were stained with hematoxylin, mounted, and examined. All histological pictures were scanned under a light microscope and analyzed by ImageJ software [29].

### 4.4. Immunofluorescence

CFs grown on glass slides were treated as indicated, fixed with paraformaldehyde (4%; *m*/*v*), and blocked with PBS containing BSA (1%, *m*/*v*) and Triton X-100 (0.1%; *m*/*v*). To detect the cytoskeleton, the cells were probed by rhodamine-phalloidin (Sigma), and nuclei were stained with 4,6-diamidino-2-phenylindole dihydrochloride (DAPI; Sigma). To detect Col-I and Col-III expression, the cells were probed with antibodies against Col-I/III overnight, followed by incubation with Alexa Fluor 555-conjugated secondary antibody (Sigma) and DAPI. The fluorescence images were captured by a Zeiss LSM780 confocal microscope (Jena, Germany) and analyzed via ImageJ.

### 4.5. Western Blot

Equal amounts of protein were separated by SDS-PAGE, wet-transferred, and blocked as previously reported [30]. The protein bands were then probed with primary antibodies (Cell Signaling) as indicated, followed by detection with horseradish peroxidase (HRP)-conjugated secondary antibodies (Sigma). Chemiluminescence images were observed using SuperSignal chemiluminescent substrate (Thermo Fisher; Waltham, MA, USA) with a ChemiDoc™ Touch Imaging System (Bio-Rad, Hercules, CA, USA). Protein ratios were calculated using ImageJ by normalizing to the densities of GAPDH and the control group. With exceptions, the internal control of phosphorylated protein was replaced with its own total protein if the latter showed no response to treatments.

### 4.6. Colocalization

CFs grown on glass slides were treated with AngII (1 μM) and/or TP (10 μg/L) for 24 h. After fixing and blocking the cells, NLRP3 was probed using a mouse anti-NLRP3 antibody (Cell Signaling), followed by an Alexa Fluor 488-conjugated secondary antibody (Sigma; green). ASC or caspase-1 was probed with the corresponding rabbit primary antibodies (Cell Signaling), followed by an Alexa Fluor 555-conjugated secondary antibody (Sigma; red). The nuclei were stained with DAPI (blue). Confocal imaging was used to observe the intracellular distribution of NLRP3 and the colocalization of green and red fluorescence. Pearson’s correlation coefficient (Rr) of NLRP3 and ASC or of NLRP3 and caspase-1 was calculated using ImageJ.

### 4.7. Immunoprecipitation

3’-flag-tagged *Nlrp3* (GenBank No. BC116174) was synthesized and subcloned into pcDNA3.1 (Thermo Fisher) between *BamH*I and *EcoR*I. The DNA sequence of the construct was verified via sequencing. CFs were grown in dishes until they reached 70% confluence, and then were transfected with Flag-*Nlrp3* recombinant plasmid for 8 h using Lipofectamine2000 (Thermo Fisher) according to the manual. After 16 h of culture in complete medium, the cells were treated as indicated. IP was performed according to the manual for Protein A/G Magnetic Beads (Thermo Fisher). Briefly, the cell lysate was extracted using the Cell Lysis-Wash buffer that was supplied in the kit, and 1 mg of each protein sample was incubated with anti-Flag (Sigma) at 4 °C overnight. Immune complexes were collected using Protein A/G beads and were subjected to Western blot as described above. ImageJ was employed to calculate the protein ratio of Flag-NLRP3-associated ASC or pro-caspase-1.

### 4.8. Statistics

Data are expressed as the mean ± standard error of mean (SEM). The statistical significance of the differences between groups was determined by student’s *t*-test. *p* values less than 0.05 were considered statistically significant, and those less than 0.01 were considered highly statistically significant.
